# Genome-Wide Association Study of Morpho-Physiological Traits in *Aegilops tauschii* to Broaden Wheat Genetic Diversity

**DOI:** 10.3390/plants10020211

**Published:** 2021-01-22

**Authors:** Mazin Mahjoob Mohamed Mahjoob, Yasir Serag Alnor Gorafi, Nasrein Mohamed Kamal, Yuji Yamasaki, Izzat Sidahmed Ali Tahir, Yoshihiro Matsuoka, Hisashi Tsujimoto

**Affiliations:** 1 United Graduate School of Agricultural Sciences, Tottori University, Tottori 680-8553, Japan; m.mahjoob20@gmail.com; 2 Agricultural Research Corporation, Wheat Research Program, P.O. Box 126, Wad Medani, Sudan; yasirserag@tottori-u.ac.jp (Y.S.A.G.); renokamal@gmail.com (N.M.K.); izzatahir@yahoo.com (I.S.A.T.); 3 Arid Land Research Center, Tottori University, Tottori 680-0001, Japan; yujyamas@tottori-u.ac.jp; 4 Department of Bioscience, Fukui Prefectural University, Eiheiji, Yoshida, Fukui 910-1195, Japan; matsuoka@fpu.ac.jp

**Keywords:** *Aegilops tauschii*, *Triticum aestivum*, morpho-physiological diversity, genetic diversity, GWAS, DArTseq marker, dryland

## Abstract

*Aegilops tauschii*, the D-genome donor of bread wheat, is a storehouse of genetic diversity that can be used for wheat improvement. This species consists of two main lineages (TauL1 and TauL2) and one minor lineage (TauL3). Its morpho-physiological diversity is large, with adaptations to a wide ecological range. Identification of allelic diversity in *Ae. tauschii* is of utmost importance for efficient breeding and widening of the genetic base of wheat. This study aimed at identifying markers or genes associated with morpho-physiological traits in *Ae. tauschii*, and at understanding the difference in genetic diversity between the two main lineages. We performed genome-wide association studies of 11 morpho-physiological traits of 343 *Ae. tauschii* accessions representing the entire range of habitats using 34,829 DArTseq markers. We observed a wide range of morpho-physiological variation among all accessions. We identified 23 marker–trait associations (MTAs) in all accessions, 15 specific to TauL1 and eight specific to TauL2, suggesting independent evolution in each lineage. Some of the MTAs could be novel and have not been reported in bread wheat. The markers or genes identified in this study will help reveal the genes controlling the morpho-physiological traits in *Ae. tauschii*, and thus in bread wheat even if the plant morphology is different.

## 1. Introduction

*Aegilops tauschii* Coss. (syn. *Ae. squarrosa* auct. non L.), a wild diploid self-pollinating species (2*n* = 2*x* = 14, DD), is the D-genome donor of hexaploid bread wheat (*Triticum aestivum* L.; 2*n* = 6*x* = 42, AABBDD). It is native to Central Asia throughout the Caspian Sea region and China. About 10,000 years ago, natural hybridization between tetraploid wheat and *Ae. tauschii* [1,2,3] led to the formation of hexaploid wheat [4,5]. Only a few *Ae. tauschii* lines from a limited area were involved in this hybridization [6]. This has resulted in a narrow genetic base of the wheat D-genome during the evolution of bread wheat. This fact has been confirmed by various studies, and indicates that the D-genome of wheat has narrow genetic diversity compared with the A and B genomes [3,7,8]. However, much greater genetic diversity is present in the wild D-genome donor [9]. It is believed that *Ae. tauschii* is an excellent source of genes to widen the narrow genetic base of bread wheat, such as for drought and heat-stress tolerance [10,11]. To use the genetic diversity in *Ae. tauschii* effectively, a precise genomic and morpho-physiological analysis is needed.

The genome-wide association study (GWAS) is a leading approach to the dissection of complex traits and the detection of novel and superior alleles for crop breeding. GWAS has been used to untangle the genetic architecture of numerous traits in different crops [12,13]. Many studies have focused on understanding the genetic and morphological diversity of *Ae. tauschii* germplasm [9,14,15,16,17,18,19,20]. However, only a few studies in *Ae. tauschii* have used GWAS, focusing on cadmium stress [21], phosphorus deficiency [22], grain architecture [23], grain micronutrient concentrations [24], or other morphological traits [25]. Here, we investigated marker–trait associations (MTAs) of morpho-physiological traits that could contribute greatly to improving yield and stress adaptation in bread wheat through GWAS, and sought specific MTAs to define the sources of evolution in two of its three lineages, TauL1 and TauL2.

## 2. Results

### 2.1. Morpho-Physiological Variation

**Figure 1:**
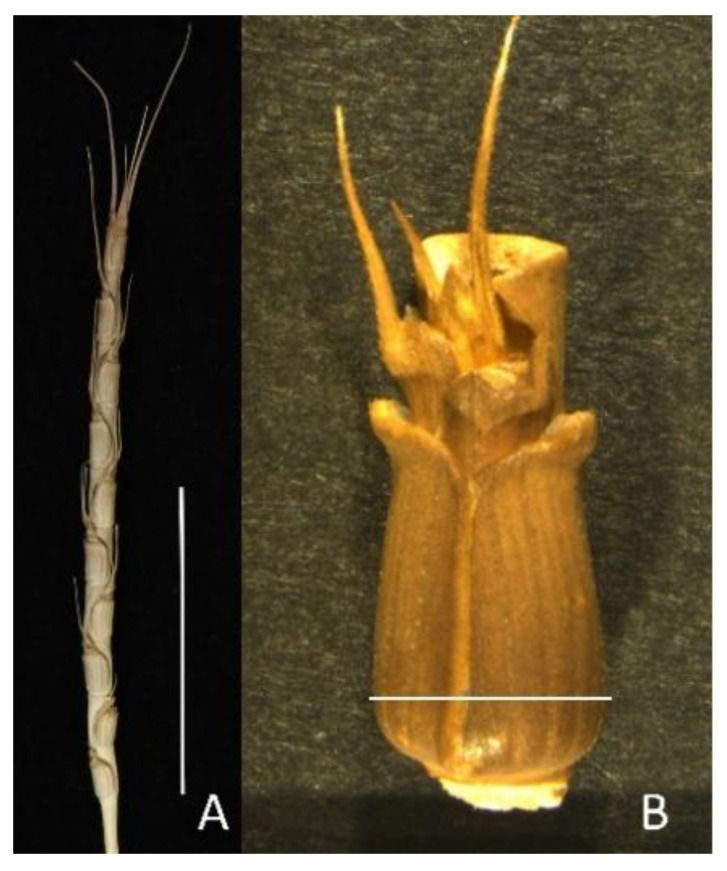
Methodology of spike measurements in *Ae. tauschii*. (**A**) Spike length was measured from the base of the lowest spikelet to the top of the highest spikelet. (**B**) Spike width was measured from the widest part of the spikelet.

**Figure 2:**
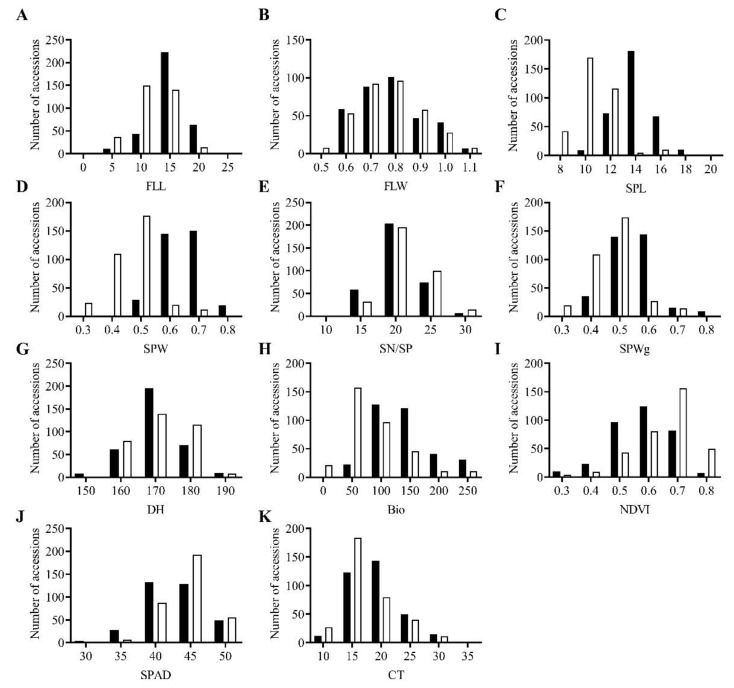
Morpho-physiological variation in *Aegilops tauschii* accessions in ■ season 1 and □ season 2. (**A**) FLL, flag leaf length; (**B**) FLW, flag leaf width; (**C**) SPL, spike length; (**D**) SPW, spike width; (**E**) SN/SP, seed number per spike; (**F**) SPWg, spike weight; (**G**) DH, days to heading; (**H**) Bio, biomass weight; (**I**) NDVI, normalized difference vegetative index; (**J**) SPAD, chlorophyll content; (**K**) CT, canopy temperature.

We studied eight morphological traits (FLL, FLW, SPL, SPW, SN/SP, SPWg, DH, and Bio) and three physiological traits (NDVI, SPAD, and CT). Spike length and width measurement methodology shown in Figure 1. ANOVA revealed high genetic variation among all accessions in all traits (Table 1 and Figure 2).

The effect of seasonal difference (S) was significant (*p* < 0.05) for all traits except for FLW and DH. The effect of genotype × seasonal difference interaction (G × S) was significant for DH, Bio, NDVI, SPAD, and CT. Morpho-physiological variations among accessions in each trait were confirmed by range, mean, standard deviation, and coefficient of variation. The coefficient of variation ranged from 4.6% to 35.5% in S1 and from 4.4% to 57.9% in S2. Heritability values were higher in morphological traits (>0.90; FLL, FLW, SPL, and SPW) than in physiological traits (<0.60; NDVI, SPAD, and CT; Table 1).

### 2.2. Correlation of Morpho-Physiological Traits in TauL1, TauL2, and All Accessions

In TauL1 and TauL2, we analyzed correlations among morpho-physiological traits (Table 2 and Table 3). Both lineages had significant positive correlations between SPWg and SPW (*r* = 0.781 in TauL1, *r* = 0.907 in TauL2), DH and Bio (*r* = 0.631 and 0.574), and SPL and SN/SP (*r* = 0.497 and 0.564). Both had negative correlations between CT and NDVI (*r* = −0.439 and −0.324), and CT and Bio (*r* = −0.427 and −0.163) (Table 2 and Table 3).

The correlations between spike-related traits (SPL, SPW, SN/SP, and SPWg) were slightly higher in TauL2 accessions than in TauL1 accessions.

We also analyzed correlations in all accessions combined (TauL1, TauL2, and TauL3) (Table 4). We found positive correlations between SPWg and SPW (*r* = 0.843), DH and Bio (*r* = 0.594), SPL and SN/SP (*r* = 0.536), FLL and FLW (*r* = 0.483), and NDVI and Bio (*r* = 0.457). We found negative correlations between CT and NDVI (*r* = −0.388), and CT and Bio (*r* = −0.304).

### 2.3. GWAS in TauL1 and TauL2 to Reveal Allelic Diversity in Each Lineage

**Figure 3:**
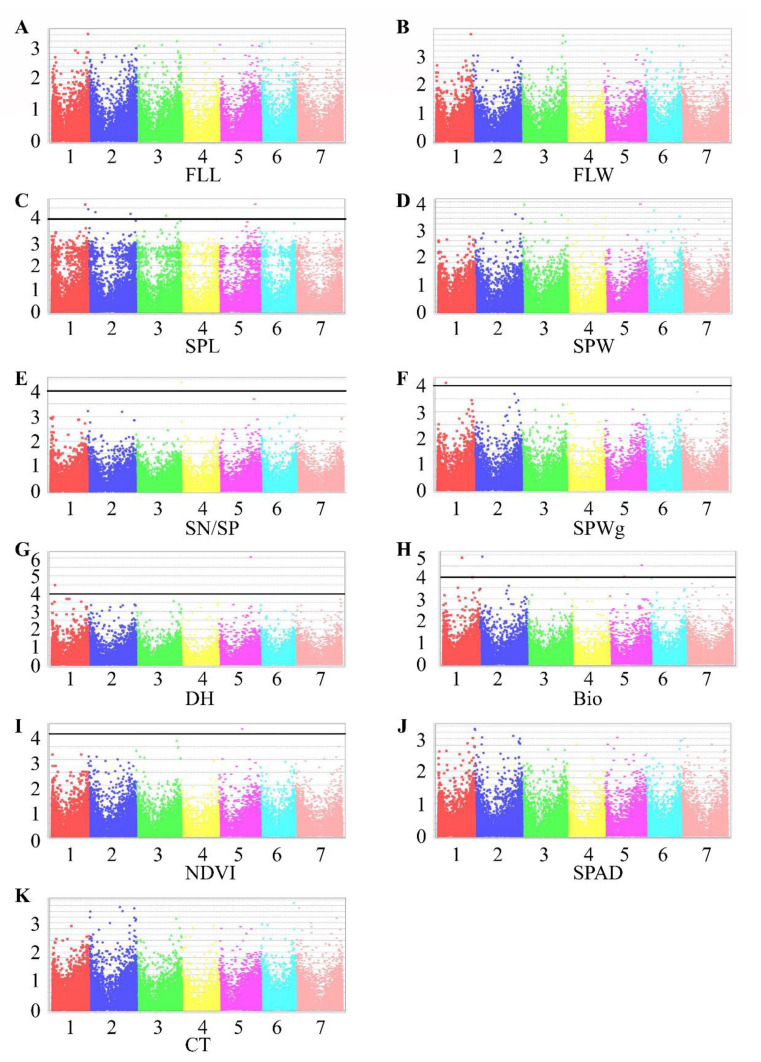
Manhattan plots representing seven chromosomes carrying significant markers detected by Mixed Linear Model using BLUP values in TauL1. (**A**) FLL, flag leaf length; (**B**) FLW, flag leaf width; (**C**) SPL, spike length; (**D**) SPW, spike width; (**E**) SN/SP, seed number per spike; (**F**) SPWg, spike weight; (**G**) DH, days to heading; (**H**) Bio, biomass weight; (**I**) NDVI, normalized difference vegetative index; (**J**) SPAD, chlorophyll content; (**K**) CT, canopy temperature. Genomic coordinates are displayed along the *X*-axis, with the negative logarithm of the association *p*-value for each single nucleotide polymorphism (SNP) displayed on the *Y*-axis, meaning that each dot on the Manhattan plot signifies a SNP. Black rules indicate the significance threshold.

**Figure 4:**
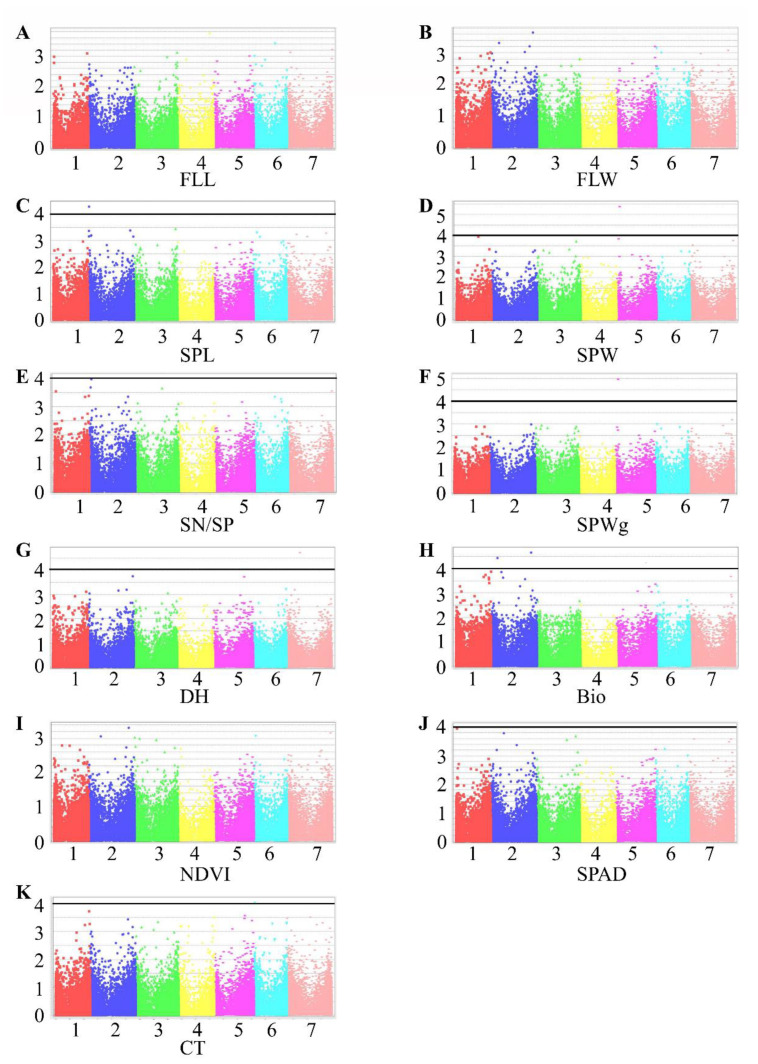
Manhattan plots representing seven chromosomes carrying significant markers detected by Mixed Linear Model using BLUP values in TauL2. (**A**) FLL, flag leaf length; (**B**) FLW, flag leaf width; (**C**) SPL, spike length; (**D**) SPW, spike width; (**E**) SN/SP, seed number per spike; (**F**) SPWg, spike weight; (**G**) DH, days to heading; (**H**) Bio, biomass weight; (**I**) NDVI, normalized difference vegetative index; (**J**) SPAD, chlorophyll content; (**K**) CT, canopy temperature. Genomic coordinates are displayed along the *X*-axis, with the negative logarithm of the association *p*-value for each single nucleotide polymorphism (SNP) displayed on the *Y*-axis, meaning that each dot on the Manhattan plot signifies a SNP. Black rules indicate the significance threshold.

GWAS revealed 15 MTAs in TauL1 and eight in TauL2 (Figure 3 and Figure 4; Table 5). TauL1 had six MTAs for SPL; four for Bio; two for DH; and one for each SN/SP, SPWg, and NDVI (Figure 3 and Table 5).

*R*^2^ values ranged from 0.10 to 0.15 and were higher than those of the significant markers in all accessions combined (0.05–0.09; Table 6). TauL2 had 1 MTA for each of SPL, SPW, SN/SP, SPWg, DH, Bio, SPAD, and CT, with *R*^2^ from 0.12 to 0.17 (Figure 4 and Table 5).

Among the MTAs detected for DH in all accessions combined, marker 32782428|F|0-17, on chromosome 5D, was detected in TauL1 also, where it had pleiotropic effects on DH and Bio (Table 5 and Table 6). All other significant MTAs differed between all accessions combined, TauL1 and TauL2. Marker 32740588, detected in TauL2, had a pleiotropic effect on SPW and SPWg. An MTA for CT was detected only in TauL2 (Figure 4 and Table 5). TauL1 and TauL2 had no MTAs in common. TauL2 had fewer MTAs than TauL1.

### 2.4. GWAS in All Accessions of Aegilops tauschii

**Figure 5:**
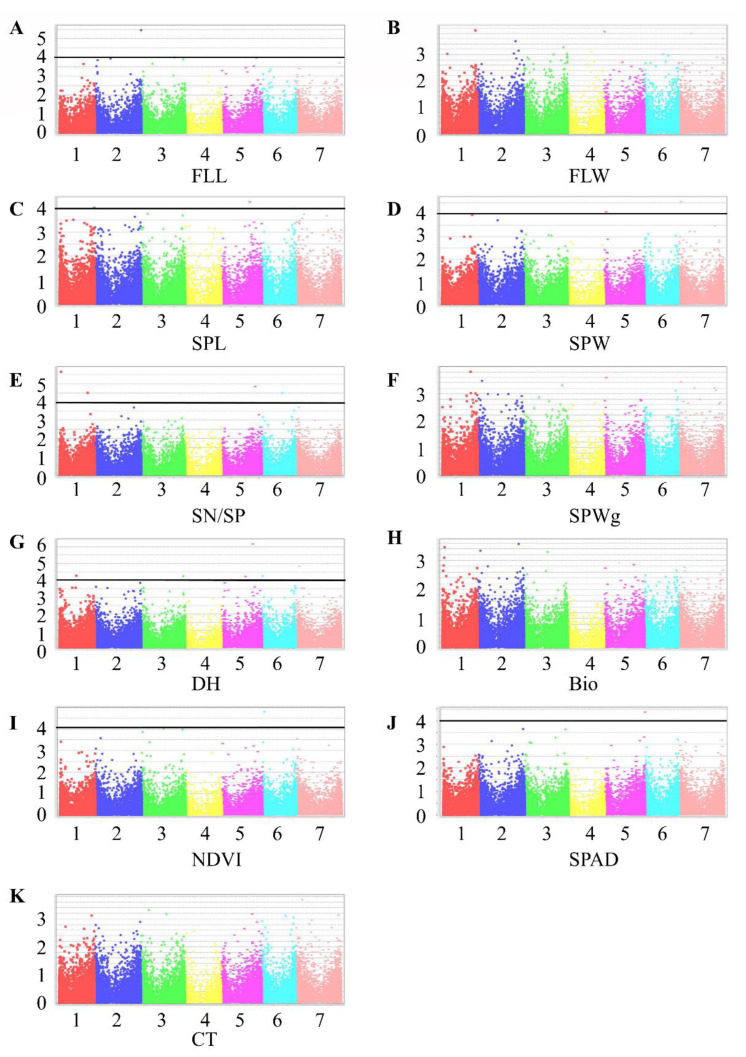
Manhattan plots representing seven chromosomes carrying the significant markers detected by Mixed Linear Model using BLUP values in all accessions. (**A**) FLL, flag leaf length; (**B**) FLW, flag leaf width; (**C**) SPL, spike length; (**D**) SPW, spike width; (**E**) SN/SP, seed number per spike; (**F**) SPWg, spike weight; (**G**) DH, days to heading; (**H**) Bio, biomass weight; (**I**) NDVI, normalized difference vegetative index; (**J**) SPAD, chlorophyll content; (**K**) CT, canopy temperature. Genomic coordinates are displayed along the *X*-axis, with the negative logarithm of the association *p*-value for each single nucleotide polymorphism (SNP) displayed on the *Y*-axis, meaning that each dot on the Manhattan plot signifies a SNP. Black rules indicate the significance threshold.

GWAS in all 343 accessions identified 23 MTAs: three each for FLL, SPL, SPW, NDVI; four for SN/SP; six for DH; and one for SPAD (Figure 5 and Table 6). *R*^2^ values ranged from 0.05 to 0.09. Most of these MTAs were different from those in TauL1 and TauL2. The one exception, 32782428|F|0-17, for DH, appeared also in TauL1 as an MTA for DH and Bio. Most of the MTAs contributed less to variability (*R*^2^) than those in TauL1 and TauL2.

### 2.5. Candidate Gene Identification

We searched for candidate genes for the MTAs in TauL1 and TauL2 (Supplementary Tables S1 and S2) and identified the possible functions. The functions show that the MTAs found here play an important role in plant adaptation and survival.

## 3. Discussion

### 3.1. Morpho-Physiological Variation in Aegilops tauschii

Among the wild species in the tribe Triticeae, *Ae. tauschii* is considered the most suitable for the genetic enhancement of wheat. The diversity of the D-genome of *Ae. tauschii* is much larger than that of hexaploid wheat’s D genome. The *Ae. tauschii* genome contains many useful genes for resistance to biotic and abiotic stresses and for seed storage proteins [26,27,28,29]. The 343 *Ae. tauschii* accessions analyzed showed significant variation in most traits studied. Spike and leaf traits had higher heritabilities than physiological traits (CT, SPAD, and NDVI) (Table 1), indicating that environmental factors greatly influence physiological traits. As spike and leaf traits are genetically determined, they are less influenced by the environment (Table 1). Selection of highly heritable traits will be effective for widening the genetic base of wheat diversity [30]. Highly correlated traits are likely to be inherited together, widening the genetic base. A positive correlation between SPW and SPWg (*r* = 0.781 in TauL1, *r* = 0.907 in TauL2, *r* = 0.843 in all accessions; Table 2, Table 3 and Table 4) indicates that an increase in SPW increases SPWg. SPW had a greater effect on grain weight than SPL. On average, grains in TauL2 were heavier and larger. Moderate to strong correlations between grain weight and size in wheat have been reported [31]. A mutation in *TaGW2-A1* increased both grain width and length in tetraploid and hexaploid wheat, which increased 1000-grain weight [32]. The correlation between SPW and SPWg was highest in TauL2 (*r* = 0.907; Table 3), indicating that TauL2 is a more suitable source for improving grain weight. A positive correlation between SPL and SN/SP indicates that an increase in SPL increases SN/SP. SPL thus affects kernel number per spike and plays an essential role in improving wheat yield [33]. Moreover, the number of grains per m^2^ and grain weight are the most important traits for determining grain yield [23].

Among physiological traits, a significant positive correlation of NDVI with Bio indicates that an increase in NDVI enhances Bio production and subsequently plant production and adaptation. The negative correlation between CT and Bio indicates that a decrease in CT increases Bio. In other words, plants with better cooling capacity will maintain better Bio. A positive correlation of DH with Bio indicates that a longer vegetative period is preferable for a higher Bio, if the environment is favorable (Table 2, Table 3 and Table 4).

### 3.2. GWAS of Morpho-Physiological Traits in TauL1 and TauL2

GWAS revealed that MTAs of morpho-physiological traits differed in both chromosome name and location between TauL1 and TauL2 (Table 5). These findings indicate that the traits have evolved independently in each lineage.

TauL1 had more MTAs for SPL, DH, and Bio than TauL2 (Figure 3 and Figure 4), indicating higher variation in these traits in TauL1. We found candidate genes in TauL1, but not in TauL2, that increase Bio and promote flowering, indicating that TauL1 is a better source for mining genes related to Bio, DH, and SPL (Supplementary Tables S1 and S2).

MTAs for CT and SPAD were found only in TauL2. As most of the accessions in TauL2 originated from Northern Iran, which has a warm and mild environment, we can speculate that these two traits contribute to the adaptation of these accessions to their habitats. Conversely, NDVI was found only in TauL1. TauL1 could be a source for NDVI gene mining, whereas TauL2 could be a source for CT and SPAD gene mining.

Mahjoob et al.’s unpublished study found that spike traits are potentially useful for differentiating between TauL1 and TauL2: SPL, SPW, and SPWg all differed significantly. In TauL1, no significant MTA was detected for SPW, and the marker *R*^2^ for SPWg was lower in TauL1 than in TauL2. These results support our conclusion that TauL2 has more diversity in SPW and SPWg than TauL1. Moreover, the SPW and SPWg candidate genes *TraesCS5D02G042200* and *TraesCS5D02G041500*, identified in TauL2, are orthologous to *Arabidopsis thaliana AT2G03590*, which encodes a transmembrane transporter that increases nitrogen fixation and promotes seed development [34]. Thus, TauL2 could be an essential source of genes related to these two traits.

### 3.3. GWAS of Morpho-Physiological Traits in All Accessions

The phenotypic contribution of markers revealed by GWAS was lower in all accessions than in TauL1 and TauL2 (Table 6). These may relate to the difference in population structures, which reduced the contribution of markers to phenotypic variation (*R*^2^).

### 3.4. Candidate Genes Revealed by GWAS in Aegilops tauschii

We found several MTAs and candidate genes associated with specific functions that play an important role in plant growth and survival. This study is the first study to use GWAS analysis of many morphological and physiological traits in *Ae. tauschii* of important agronomic value to wheat breeding, though Liu et al. [25] conducted GWAS in *Ae. tauschii* in which traits, SPL, FLL, and FLW are common. Liu et al. [25] identified 18 MTAs for only 10 of the 29 traits studied. Our study identified more MTAs, with higher R^2^ values (0.5–0.17) than most of those studied before [25] because we used GWAS for two lineages independently with more molecular markers.

### 3.5. Marker Traits Revealed in Wheat from Aegilops tauschii

To study the usefulness of the markers revealed in *Ae. tauschii* and their appearance in wheat, we reviewed previous GWAS studies of wheat (Table 7). Li et al. (2019), Ward et al. (2019), and Jamil et al. (2019) [35,36,37] reported several MTAs for DH, FLL, SN/SP, and SPL on different chromosomes.

We found MTAs for DH on chromosomes 1D, 5D, and 7D also found by Lie et al. [35]. We identified novel MTAs on chromosomes 3D and 6D for DH; on 2D, 3D, and 5D for FLL; on 1D, 2D, 5D, and 6D for SN/SP; and on 1D, 2D, 3D, 5D, and 6D for SPL. In TauL1, we found novel MTAs on 1D and 5D for DH; on 4D for SN/SP; and on 1D, 2D, 3D, and 5D for SN/SP. In TauL2 (which supplied the D-genome of hexaploid wheat [38]), we identified three novel MTAs: two on 2D associated with SN/SP and SPL, and one on 7D associated with DH. Those MTAs can be easily transferred to the D-genome of wheat where they would be expected to increase yield. Markers on 7D associated with DH can be transferred to improve early flowering in later-flowering variants, especially in drylands.

## 4. Materials and Methods

### 4.1. Plant Materials

We used 343 *Ae. tauschii* accessions representing the entire range of natural habitats (Supplementary Table S3). These comprised AE accessions from the Institut für Pflanzengenetik und Kulturpflanzenforschung, Germany; AT accessions from the Faculty of Agriculture, Okayama University, Japan; CGN accessions from the Instituut Voor Planten Veredeling, Landbouwhoge School, Wageningen, the Netherlands; IG accessions from the International Center for Agricultural Research in the Dry Areas, Syria; KU accessions from the Germplasm Institute, Faculty of Agriculture, Kyoto University, Japan; and PI accessions from the US Department of Agriculture. Within the panel, 182 accessions belong to TauL1, 156 to TauL2, and 5 to TauL3 (Supplementary Table S3).

### 4.2. Morpho-Physiological Evaluation

Details of the morpho-physiological evaluations and data collection are summarized in Table 8. Spike length and width were measured using ruler as shown in Figure 1. All accessions were characterized in the research field of the Arid Land Research Center, Tottori University (Tottori, Japan; 35°32′ N, 134°13′ E), during the winter–spring seasons of 2016–17 (S1) and 2017–18 (S2), in an augmented complete block design with three checks selected randomly. We measured 11 morpho-physiological traits: flag leaf length (FLL), flag leaf width (FLW), spike length (SPL), spike width (SPW), seed number per spike (SN/SP), spike weight (SPWg), days to heading (DH), biomass (Bio), normalized difference vegetative index (NDVI), canopy temperature (CT), and chlorophyll content (SPAD).

### 4.3. Statistical Analysis of Agronomic Traits

ANOVA was conducted in Plant Breeding Tools (PBTools) v. 1.4 software (International Rice Research Institute, http://bbi.irri.org/products). Using genetic variance (*V*_g_) and environmental variance (*V*_e_), we calculated broad-sense heritability [*H*^2^ = *V*_g_/(*V*_g_ + *V*_e_)] of each trait [39]. Because genotype × season interactions were significant, we estimated best linear unbiased predictions (BLUPs) for each trait. We used BLUP data for trait correlation analysis in TauL1, TauL2, and all accessions in SPSS v. 25 software [40].

### 4.4. Genotyping and Marker–Trait Association (MTA) Analysis

Genomic DNA was extracted from young leaves by using the CTAB method [41]. The DNA samples (30 µL; 50–100 ng µL^−1^) were sent to Diversity Arrays Technology Pty Ltd., Australia (http://www.diversityarrays.com), for a whole-genome scan on the DArTseq platform (DArT P/L, Canberra, Australia)). DArTseq is a genotyping-by-sequencing method which utilizes a Next-Generation Sequencing approach to sequence the most informative representations of genomic DNA samples to aid marker discovery. In total, DArTseq generates 59,193 silico and 55,390 SNP markers. We selected the markers with a call rate of 90% (10% missing data) and obtained 3117 SNP and 47,072 Sillco markers. The Fisher exact test was applied to determine if the two alleles were independent SNP markers. Single nucleotide polymorphisms (SNPs) or Silico DArT markers with a minor allele frequency of <5% were removed from the analysis. The remaining 34,829 SNPs and Silico DArT markers were used for genomic analysis.

We performed GWAS with BLUP values for each phenotype using a Mixed Linear Model (MLM) in TASSEL v. 5 software [42]. For all traits, the Bonferroni–Holm correction for multiple testing (α = 0.05) was too stringent. Thus, markers with an adjusted -log10 (*p*-value) ≥ 4.0 were regarded as significant. To search for candidate genes, we performed a BLAST search of the sequence of each significant marker against the Chinese Spring RefSeq v. 1.0 wheat reference genome (IWGSC 2020). The position where the tag hit the best match was extended by 0.5 Mb in both directions, and that sequence was then used in a BLAST search of the Ensembl *T. aestivum* database (http://plants.ensembl.org/*Triticum*_*aestivum*/Info/Index) to find predicted genes or proteins within this region. To study and validate the usefulness of the MTAs revealed in *Ae. tauschii* to wheat breeding, we compared it with previous MTAs revealed in bread wheat.

## 5. Conclusions

We conducted GWAS analysis of morpho-physiological traits in a diverse panel of *Ae. tauschii* accessions and identified several MTAs and corresponding candidate genes. Some of the candidate genes had exact functions related to the trait studied. Morphological traits are more stable and less affected by environmental factors than physiological traits. GWAS analysis revealed that morphological traits had higher number of MTAs compared to physiological traits (Table 5 and Table 6). This facilitates the use of morphological trait selection in wheat breeding through marker-assisted selection. Comparing our findings with other studies in wheat suggested that some of the MTAs and genes identified here are not present in bread wheat. Our results reveal some of the hidden diversity in *Ae. tauschii* and provide a basis for its use in wheat breeding through direct and indirect crossing [43].

The information presented here could also help explain the mechanisms controlling the morpho-physiological traits in *Ae. tauschii*, which will pave the way to a better understanding of the mechanisms in bread wheat. Multiple-synthetic-derivative wheat lines incorporate a wide range of genetic diversity of *Ae. tauschii* were developed, and heat- and drought-resistant lines were identified through the use of such lines [11,44,45]. These facts support the indispensable role of the D-genome of *Ae. tauschii* in wheat breeding for high productivity and stress adaptation.

## Figures and Tables

**Table 1:** Analysis of variance (ANOVA) of 11 morpho-physiological traits measured in 343 *Aegilops tauschii* accessions grown under field conditions during seasons 2016–17 (S1) and 2017–18 (S2).

Trait	Season	Accession Range	Mean	*p*-Value (G)	*p*-Value (S)	*p*-Value (G × S)	SED ± (G)	H^2^	CV (%)
FLL (cm)	S1	5.11–22.72	14.98	0.001			3.3292	0.96	21.2
	S2	2.78–21.66	11.89	0.1394			3.539		27.6
	BLUP	4.29–21.42	13.44	<0.001	<0.001	1	1.2933		
FLW (cm)	S1	0.41–1.14	0.80	<0.001			0.1248	0.97	17.0
	S2	0.43–1.12	0.79	<0.001			0.1259		16.3
	BLUP	0.39–1.17	0.80	<0.001	0.9996	0.9975	0.0482		
SPL (cm)	S1	9.89–18.70	13.94	<0.001			1.021	0.98	10.3
	S2	6.92–17.03	10.66	<0.001			1.0216		15.4
	BLUP	8.63 -17.76	12.30	<0.001	<0.001	0.9998	0.4564		
SPW (cm)	S1	0.46–0.76	0.62	<0.001			0.0436	0.96	10.8
	S2	0.30–0.74	0.48	<0.001			0.0386		16.5
	BLUP	0.38–0.75	0.55	<0.001	<0.001	0.8922	0.028		
SN/SP	S1	11.83–32.89	20.42	0.0156			3.128	0.89	18.3
	S2	11.29–31.29	21.50	<0.001			2.3419		17.2
	BLUP	13.15–31.83	20.95	<0.001	<0.001	0.7002	2.0166		
SPWg	S1	0.30–0.77	0.56	0.1799			0.1075	0.90	15.2
	S2	0.29–0.74	0.47	0.0225			0.0849		17.5
	BLUP	0.27–0.76	0.52	<0.001	<0.001	0.9998	0.0496		
DH	S1	134–194	170.99	<0.001			1.4354	0.86	4.6
	S2	132–196	171.76	<0.001			2.6035		4.4
	BLUP	147–195	171.39	<0.001	0.1052	<0.001	3.89		
Bio	S1	50.30–260.90	140.34	<0.001			4.862	0.78	35.5
	S2	50.30–260.40	86.35	<0.001			2.1117		57.9
	BLUP	42.53–260.59	113.51	<0.001	<0.001	<0.001	31.766		
NDVI	S1	0.30–0.79	0.58	<0.001			0.0503	0.13	17.7
	S2	0.28–0.82	0.66	<0.001			0.0087		16.8
	BLUP	0.41- 0.78	0.62	0.1323	<0.001	<0.001	0.0979		
SPAD	S1	29.10–52.40	42.82	<0.001			2.3947	0.28	10.0
	S2	33.40–52.36	44.33	<0.001			0.4336		7.9
	BLUP	33.10–51.19	43.58	0.0047	<0.001	<0.001	3.5841		
CT (°C)	S1	10.62–34.48	18.96	<0.001			1.2967	0.55	21.9
	S2	9.40- 36.90	17.52	<0.001			0.7403		26.5
	BLUP	11.14–31.73	18.25	<0.001	<0.001	<0.001	3.4195		

CV: Coefficient of variation, SED: Standard error of a difference.

**Table 2:** Morpho-physiological correlation analysis in TauL1 performed using best linear unbiased predictions (BLUPs) of two consecutive seasons (2016–17 and 2017–18).

Trait	FLL	FLW	SPL	SPW	SN/SP	SPWg	DH	Bio	NDVI	SPAD	CT
FLL		0.530 **	0.264 **	0.086	0.178*	0.174 *	−0.170 *	0.026	0.151 *	−0.085	−0.035
		0.000	0.000	0.250	0.016	0.019	0.022	0.730	0.042	0.253	0.641
FLW			0.196 **	0.241 **	0.067	0.292 **	−0.315 **	−0.088	0.092	−0.029	−0.043
			0.008	0.001	0.367	0.000	0.000	0.237	0.219	0.694	0.565
SPL				0.049	0.497 **	−0.014	0.183 *	0.134	0.271 **	−0.060	−0.209 **
				0.510	0.000	0.851	0.014	0.071	0.000	0.417	0.005
SPW					−0.264 **	0.781 **	−0.094	0.035	0.084	0.162 *	−0.057
					0.000	0.000	0.208	0.637	0.260	0.029	0.442
SN/SP						−0.224 **	0.239 **	0.065	0.170 *	−0.093	−0.152 *
						0.002	0.001	0.381	0.022	0.210	0.040
SPWg							−0.177 *	−0.007	0.093	0.213 **	0.011
							0.017	0.930	0.210	0.004	0.882
DH								0.631 **	0.240**	0.068	−0.286 **
								0.000	0.001	0.364	0.000
Bio									0.460 **	0.085	−0.427 **
									0.000	0.256	0.000
NDVI										−0.050	−0.439 **
										0.501	0.000
SPAD											−0.022
											0.772

Asterisks: Correlation is significant at * 0.05 or ** 0.01 level. Upper values are correlation coefficients (*R*^2^); lower values are probabilities (*P*). Number of accessions = 182.

**Table 3:** Morpho-physiological correlation analysis in TauL2 performed using best linear unbiased predictions (BLUPs) of two consecutive seasons (2016–17 and 2017–18).

Trait	FLL	FLW	SPL	SPW	SN/SP	SPWg	DH	Bio	NDVI	SPAD	CT
FLL		0.433 **	0.254 **	0.085	0.162 *	0.124	0.005	0.181 *	0.256 **	−0.091	−0.084
		0.000	0.001	0.292	0.044	0.124	0.950	0.024	0.001	0.257	0.295
FLW			0.062	0.308 **	−0.071	0.245 **	−0.334 **	−0.097	0.053	0.128	−0.108
			0.442	0.000	0.381	0.002	0.000	0.226	0.507	0.110	0.181
SPL				−0.051	0.564 **	−0.108	0.137	0.151	0.180 *	0.052	−0.197 *
				0.525	0.000	0.181	0.088	0.060	0.025	0.515	0.014
SPW					−0.285 **	0.907 **	−0.161 *	0.101	0.228 **	0.019	−0.096
					0.000	0.000	0.044	0.208	0.004	0.818	0.231
SN/SP						−0.260 **	0.189 *	0.063	0.004	0.005	−0.167 *
						0.001	0.018	0.434	0.963	0.946	0.037
SPWg							−0.106	0.083	0.222 **	0.001	−0.063
							0.186	0.303	0.005	0.990	0.432
DH								0.574 **	0.213 **	0.046	−0.003
								0.000	0.008	0.566	0.970
Bio									0.457 **	−0.003	−0.163 *
									0.000	0.968	0.042
NDVI										−0.003	−0.324 **
										0.974	0.000
SPAD											−0.116
											0.148

Asterisks: Correlation is significant at * 0.05 or ** 0.01 level. Upper values are correlation coefficients; lower values are probabilities (*P*). Number of accessions = 156.

**Table 4:** Morpho-physiological correlation analysis in *Aegilops tauschii* performed using best linear unbiased predictions (BLUPs) of two consecutive seasons (2016–17 and 2017–18).

Trait	FLL	FLW	SPL	SPW	SN/SP	SPWg	DH	Bio	NDVI	SPAD	CT
FLL		0.483 **	0.268 **	0.088	0.176 **	0.155 **	−0.101	0.093	0.192 **	−0.092	−0.047
		0.000	0.000	0.105	0.001	0.004	0.061	0.085	0.000	0.088	0.390
FLW			0.126 *	0.269 **	−0.001	0.265 **	−0.331 **	−0.088	0.083	0.047	−0.074
			0.020	0.000	0.986	0.000	0.000	0.102	0.125	0.383	0.172
SPL				0.005	0.536 **	−0.050	0147 **	0.140 **	0.219 **	−0.022	−0.183 **
				0.933	0.000	0.352	0.006	0.009	0.000	0.683	0.001
SPW					−0.269 **	0.843 **	−0.129 *	0.066	0.148 **	0.092	−0.073
					0.000	0.000	0.017	0.223	0.006	0.088	0.179
SN/SP						−0.236 **	0.206 **	0.065	0.090	−0.055	−0.149 **
						0.000	0.000	0.232	0.097	0.313	0.006
SPWg							−0.144 **	0.037	0.152 **	0.107 *	−0.022
							0.007	0.489	0.005	0.048	0.680
DH								0.594 **	0.215 **	0.054	−0.156 **
								0.000	0.000	0.321	0.004
Bio									0.457 **	0.042	−0.304 **
									0.000	0.435	0.000
NDVI										−0.025	−0.388 **
										0.651	0.000
SPAD											−0.068
											0.209

Asterisks: Correlation is significance at * 0.05 or ** 0.01 level. Upper values are correlation coefficients; lower values are probabilities (*P*).

**Table 5:** Marker–trait associations in TauL1 and TauL2 revealed by DArTseq markers.

Lineage	Trait	Marker	Chromo-Some	Marker (*R*^2^)	SNPs	Desirable Effect Alleles	Contribution of 1st Allele	Contribution of 2nd Allele
TauL1	SPL	32763608|F|0-15	1D	0.15	A/G	G	−5E+00	−5E+00
	SPL	32743501|F|0-5	2D	0.13	A/G	A	−5E+00	−5E+00
	SPL	32765113|F|0-56	2D	0.13	C/G	G	−4E+00	−4E+00
	SPL	32784018|F|0-39	2D	0.12	C/T	C	−8E+00	−5E+00
	SPL	32745140|F|0-54	3D	0.12	A/G	G	−4E+00	−6E+00
	SPL	32740085|F|0-47	5D	0.12	A/G	A	−8E+00	−5E+00
	SN/SP	32774197|F|0-39	4D	0.14	A/T	A	3E+00	−9E+00
	SPWg	32731844	1D	0.10	A/C	A	7E−02	0E+00
	DH	32722593	1D	0.13	A/C	A	1E+01	0E+00
	DH	32782428|F|0-17	5D	0.15	C/T	C	−2E+01	0E+00
	Bio	32750474	1D	0.12	A/C	A	−6E+01	0E+00
	Bio	32755747	2D	0.12	A/C	A	−5E+01	0E+00
	Bio	32782428|F|0-17	5D	0.11	C/T	C	−1E+02	0E+00
	Bio	32732820	5D	0.11	A/C	A	5E+01	0E+00
	NDVI	32787209|F|0-56	5D	0.12	A/G	A	1E−01	4E−02
TauL2	SPL	32777153	2D	0.14	A/C	A	5E+00	0E+00
	SPW	32740588	5D	0.17	A/C	A	1E−01	0E+00
	SN/SP	32746301|F|0-43	2D	0.14	C/G	G	−6E+00	−1E+01
	SPWg	32740588	5D	0.16	A/C	A	1E−01	0E+00
	DH	32764424	7D	0.14	A/C	A	−1E+01	0E+00
	Bio	4291519	2D	0.13	A/C	A	−5E+01	0E+00
	SPAD	32785603	1D	0.13	A/C	A	4E+00	0E+00
	CT	32786555	6D	0.12	A/C	A	3E+00	0E+00

The desirable allele is that with a greater contribution to phenotypic variation.

**Table 6:** Marker–trait associations in all accessions combined revealed by DArTseq markers.

Lineage	Trait	Marker	Chromo-Some	Marker (*R*^2^)	SNPs	Desirable Effect Alleles	Contribution of 1st Allele	Contribution of 2nd Allele
All accessions combined	FLL	32723781	2D	0.08	A/C	A	−1E−01	0E+00
	FLL	32761831|F|0-30	3D	0.06	C/T	C	−5E−01	3E−02
	FLL	32765433|F|0-21	5D	0.06	C/T	C	−5E−01	8E−03
	SPL	4323996|F|0-42	5D	0.06	C/T	T	−3E−02	−6E−02
	SPL	32770344|F|0-19	1D	0.06	C/T	C	−2E−01	−1E−01
	SPL	4321487|F|0-67	6D	0.06	A/C	A	−1E−01	−1E−01
	SPW	32777197	7D	0.06	A/C	A	6E−02	0E+00
	SPW	32749969	1D	0.05	A/C	A	5E−02	0E+00
	SPW	32768546	5D	0.05	A/C	A	3E−02	0E+00
	SN/SP	32776149	1D	0.07	A/C	A	−7E−02	0E+00
	SN/SP	32787577|F|0-20	5D	0.07	C/T	T	−9E−03	−8E−02
	SN/SP	32719260	6D	0.06	A/C	A	4E−02	0E+00
	SN/SP	32782749	1D	0.06	A/C	A	4E−02	0E+00
	DH	32782428|F|0-17	5D	0.09	C/T	C	−2E−02	2E−02
	DH	32786608|F|0-9	7D	0.07	C/G	C	7E−02	6E−02
	DH	32778284	5D	0.05	A/C	A	3E−02	0E+00
	DH	32728973	3D	0.05	A/C	A	−5E−02	0E+00
	DH	32760744|F|0-62	6D	0.05	C/T	C	7E−02	7E−02
	DH	32756563	1D	0.05	A/C	A	4E−02	0E+00
	NDVI	32756802	6D	0.06	A/C	A	6E−02	0E+00
	NDVI	32780727	3D	0.05	A/C	A	5E−02	0E+00
	NDVI	32732406	3D	0.05	A/C	A	7E−02	0E+00
	SPAD	32778541	5D	0.06	A/C	A	2E−02	0E+00

The desirable allele is that with a greater contribution to phenotypic variation.

**Table 7:** Comparison of MTAs in bread wheat reported previously and those identified in this study in *Aegilops tauschii.*

Reference	Species	Trait	Chromosome						
			1D	2D	3D	4D	5D	6D	7D
Li et al. (2019)	*T. aestivum*	DH							
Ward et al. (2019)	*T. aestivum*	DH		**x**					**x**
Jami et al. (2019)	*T. aestivum*	DH	**x**				**x**		**x**
Current study	TauL1	DH	**x**				**x**		
Current study	TauL2	DH							**x**
Current study	All	DH	**x**		**x**		**x**	**x**	**x**
Li et al. (2019)	*T. aestivum*	FLL							**x**
Current study	TauL1	FLL							
Current study	TauL2	FLL							
Current study	All	FLL		**x**	**x**		**x**		
Li et al. (2019)	*T. aestivum*	FLW							
Current study	TauL1	FLW							
Current study	TauL2	FLW							
Current study	All	FLW							
Ward et al. (2019)	*T. aestivum*	SN/SP				**x**			
Current study	TauL1	SN/SP				**x**			
Current study	TauL2	SN/SP		**x**					
Current study	All	SN/SP	**x**				**x**	**x**	
Li et al. (2019)	*T. aestivum*	SPL							**x**
Current study	TauL1	SPL	**x**	**x**	**x**		**x**		
Current study	TauL2	SPL		**x**					
Current study	All	SPL	**x**				**x**	**x**	

Bold x: Marker identified in previous studies.

**Table 8:** Morpho-physiological traits measured, their abbreviations, and definitions.

Trait	Abbreviation	Measurement/Definition
Flag leaf length	FLL (cm)	Measured from three tillers of each accession.
Flag leaf width	FLW (cm)	Measured from three tillers of each accession.
Spike length	SPL (cm)	Measured at the middle spike after maturity stage in five spikes.
Spike width	SPW (cm)	Measured at the middle of five spikes after maturity stage in five spikes.
Seed number/Spike	SN/SP	Counted from five spikes at harvesting.
Seed weight/Spike	SPWg (g)	Measured using five spikes one from each tiller using a sensitive scale.
Days to heading	DH	Recorded when the whole spike above the flag leaf position fully emerged on the earliest tiller in each plant of each accession.
Biomass weight	Bio (g)	Measured after harvesting and drying in a glasshouse from five plants were counted.
Normalized Difference Vegetation Index	NDVI	A vegetative index that compares reflectance in the red and near infrared regions. Measured during flowering using a handheld optical sensor unit (Green Seeker), 2012 NTech Industries, Inc., Ukiah, CA, USA.
Canopy temperature	CT (°C)	Measured during flowering using an inferred thermometer AD-5611A.
Chlorophyll content	SPAD	Measured at the flowering stage from the middle of the flag leaf of three tillers using A Minolta brand chlorophyll meter (Model SPAD-502; Spectrum Technologies Inc. Plainfield, IL).

## Data Availability

This study did not report any data.

## Supplementary Materials

The following are available online at https://www.mdpi.com/2223-7747/10/2/211/s1, Supplementary Table S1: Functions of genes associated with morpho-physiological traits in TauL1 revealed by genome-wide association study (GWAS) using a Mixed Linear Model with DArTseq markers. Supplementary Table S2: Functions of genes associated with morpho-physiological traits in TauL2 revealed by genome-wide association study (GWAS) using a Mixed Linear Model with DArTseq markers. Supplementary Table S3: Plant materials used in this study.

## References

[1] Renfrew J.M. (1973). Palaeoethnobotany. The Prehistoric Food Plants of the Near East and Europe.

[2] Gill B.S., Raupp W.J. (1987). Direct genetic transfers from *Aegilops squarrosa* L. to hexaploid wheat. Crop Sci..

[3] Lubbers E.L., Gill K.S., Cox T.S., Gill B.S. (1991). Variation of molecular markers among geographically diverse accessions of *Triticum tauschii*. Genome.

[4] Kihara H. (1944). Discovery of the DD-analyser, one of the ancestors of *Triticum vulgare*. Agric. Hortic..

[5] Mcfadden E.S., Sears E.R. (1946). The origin of *triticum spelta* and its free-threshing hexaploid relatives. J. Hered..

[6] Lagudah E.S., Appels R., McNeil D. (1991). The *Nor-D3* locus of *Triticum tauschii*: Natural variation and genetic linkage to markers in chromosome 5. Genome.

[7] Kam-Morgan L.N.W., Gill B.S., Muthukrishnan S. (1989). DNA restriction fragment length polymorphisms: A strategy for genetic mapping of D genome of wheat. Genome.

[8] Akhundov I., Nevzorov V.B. (2010). A simple characterization of Student’s t3 distribution. Stat. Probab. Lett..

[9] Naghavi M.R., Aghaei M.J., Taleei A.R., Omidi M., Mozafari J., Hassani M.E. (2009). Genetic diversity of the D-genome in *T. aestivum* and *Aegilops* species using SSR markers. Genet. Resour. Crop Evol..

[10] Elbashir A.A.E., Gorafi Y.S.A., Tahir I.S.A., Kim J.S., Tsujimoto H. (2017). Wheat multiple synthetic derivatives: A new source for heat stress tolerance adaptive traits. Breed. Sci..

[11] Itam M., Abdelrahman M., Yamasaki Y., Mega R., Gorafi Y., Akashi K., Tsujimoto H. (2020). *Aegilops tauschii* introgressions improve physio-biochemical traits and metabolite plasticity in bread wheat under drought stress. Agronomy.

[12] Suwarno W.B., Pixley K.V., Palacios-Rojas N., Kaeppler S.M., Babu R. (2015). Genome-wide association analysis reveals new targets for carotenoid biofortification in maize. Theor. Appl. Genet..

[13] Sun C., Zhang F., Yan X., Zhang X., Dong Z., Cui D., Chen F. (2017). Genome-wide association study for 13 agronomic traits reveals distribution of superior alleles in bread wheat from the Yellow and Huai Valley of China. Plant Biotechnol. J..

[14] Matsuoka Y., Aghaei M.J., Abbasi M.R., Totiaei A., Mozafari J., Ohta S. (2008). Durum wheat cultivation associated with *Aegilops tauschii* in northern Iran. Genet. Resour. Crop Evol..

[15] Matsuoka Y., Nishioka E., Kawahara T., Takumi S. (2009). Genealogical analysis of subspecies divergence and spikelet-shape diversification in central Eurasian wild wheat *Aegilops tauschii* Coss. Plant Syst. Evol..

[16] Mizuno N., Yamasaki M., Matsuoka Y., Kawahara T., Takumi S. (2010). Population structure of wild wheat D-genome progenitor *Aegilops tauschii* Coss.: Implications for intraspecific lineage diversification and evolution of common wheat. Mol. Ecol..

[17] Matsuoka Y., Takumi S., Kawahara T. (2015). Intraspecific lineage divergence and its association with reproductive trait change during species range expansion in central Eurasian wild wheat *Aegilops tauschii* Coss. (Poaceae). BMC Evol. Biol..

[18] Sohail Q., Shehzad T., Kilian A., Eltayeb A.E., Tanaka H., Tsujimoto H. (2012). Development of diversity array technology (DArT) markers for assessment of population structure and diversity in *Aegilops tauschii*. Breed. Sci..

[19] Dudnikov A.J., Kawahara T. (2006). *Aegilops tauschii*: Genetic variation in Iran. Genet. Resour. Crop Evol..

[20] Nishijima R., Okamoto Y., Hatano H., Takumi S. (2017). Quantitative trait locus analysis for spikelet shape-related traits in wild wheat progenitor *Aegilops tauschii*: Implications for intraspecific diversification and subspecies differentiation. PLoS ONE.

[21] Qin P., Wang L., Liu K., Mao S., Li Z., Gao S., Shi H., Liu Y. (2015). Genomewide association study of *Aegilops tauschii* traits under seedling-stage cadmium stress. Crop J..

[22] Liu Y., Wang L., Deng M., Li Z., Lu Y., Wang J., Wei Y., Zheng Y. (2015). Genome-wide association study of phosphorus-deficiency-tolerance traits in *Aegilops tauschii*. Theor. Appl. Genet..

[23] Arora S., Singh N., Kaur S., Bains N.S., Uauy C., Poland J., Chhuneja P. (2017). Genome-Wide Association Study of Grain Architecture in Wild Wheat *Aegilops tauschii*. Front. Plant Sci..

[24] Arora S., Cheema J., Poland J., Uauy C., Chhuneja P. (2019). Genome-Wide Association Mapping of Grain Micronutrients Concentration in *Aegilops tauschii*. Front. Plant Sci..

[25] Liu Y., Wang L., Mao S., Liu K., Lu Y., Wang J., Wei Y., Zheng Y. (2015). Genome-wide association study of 29 morphological traits in *Aegilops tauschii*. Sci. Rep..

[26] Gill K.S., Lubbers E.L., Gill B.S., Raupp W.J., Cox T.S. (1991). A genetic linkage map of *Triticum tauschii* (DD) and its relationship to the D genome of bread wheat (AABBDD). Genome.

[27] Pestsova E., Korzun V., Goncharov N.P., Hammer K., Ganal M.W., Röder M.S. (2000). Microsatellite analysis of *Aegilops tauschii* germplasm. Theor. Appl. Genet..

[28] Assefa S., Fehrmann H. (2004). Evaluation of *Aegilops tauschii* Coss. for resistance to wheat stem rust and inheritance of resistance genes in hexaploid wheat. Genet. Resour. Crop Evol..

[29] Naghavi M.R., Mardi M. (2010). Characterization of genetic variation among accessions of *Aegilops tauschii*. Asia Pac. J. Mol. Biol. Biotechnol..

[30] Maniee M., Kahrizi D., Mohammadi R. (2009). Genetic variability of some morpho-physiological traits in durum wheat (*Triticum turgidum* var. Durum). J. Appl. Sci..

[31] Rasheed A., Xia X., Ogbonnaya F., Mahmood T., Zhang Z., Mujeeb-Kazi A., He Z. (2014). Genome-wide association for grain morphology in synthetic hexaploid wheats using digital imaging analysis. BMC Plant Biol..

[32] Simmonds J., Scott P., Brinton J., Mestre T.C., Bush M., del Blanco A., Dubcovsky J., Uauy C. (2016). A splice acceptor site mutation in *TaGW2-A1* increases thousand grain weight in tetraploid and hexaploid wheat through wider and longer grains. Theor. Appl. Genet..

[33] Guo Z., Chen D., Alqudah A.M., Röder M.S., Ganal M.W., Schnurbusch T. (2017). Genome-wide association analyses of 54 traits identified multiple loci for the determination of floret fertility in wheat. New Phytol..

[34] Carter A.M., Tegeder M. (2016). Increasing nitrogen fixation and seed development in soybean requires complex adjustments of nodule nitrogen metabolism and partitioning processes. Curr. Biol..

[35] Li F., Wen W., Liu J., Zhang Y., Cao S., He Z., Rasheed A., Jin H., Zhang C., Yan J. (2019). Genetic architecture of grain yield in bread wheat based on genome-wide association studies. BMC Plant Biol..

[36] Ward B.P., Brown-Guedira G., Kolb F.L., Van Sanford D.A., Tyagi P., Sneller C.H., Griffey C.A., Li F., Wen W., Liu J. (2019). Genome-wide association studies for yield-related traits in soft red winter wheat grown in Virginia. PLoS ONE.

[37] Jamil M., Ali A., Gul A., Ghafoor A., Napar A.A., Ibrahim A.M.H., Naveed N.H., Yasin N.A., Mujeeb-Kazi A. (2019). Genome-wide association studies of seven agronomic traits under two sowing conditions in bread wheat. BMC Plant Biol..

[38] Matsuoka Y., Nasuda S., Ashida Y., Nitta M., Tsujimoto H., Takumi S., Kawahara T. (2013). Genetic basis for spontaneous hybrid genome doubling during allopolyploid speciation of common wheat shown by natural variation analyses of the paternal species. PLoS ONE.

[39] Comstock R.E., Robinson H.F. Genetic parameters, their estimation and significance. Proceedings of the 6th International Grassland Congress.

[40] Gouda M.A. (2015). Common pitfalls in reporting the use of SPSS software. Med. Princ. Pract..

[41] Saghai-Maroof M.A., Soliman K.M., Jorgensen R.A., Allard R.W. (1984). Ribosomal DNA spacer-length polymorphisms in barley: Mendelian inheritance, chromosomal location, and population dynamics. Proc. Natl. Acad. Sci. USA.

[42] Bradbury P.J., Zhang Z., Kroon D.E., Casstevens T.M., Ramdoss Y., Buckler E.S. (2007). TASSEL: Software for association mapping of complex traits in diverse samples. Bioinformatics.

[43] Kishii M. (2019). An update of recent use of *Aegilops* species in wheat breeding. Front. Plant Sci..

[44] Elbashir A.A.E., Gorafi Y.S.A., Tahir I.S.A., Elhashimi A.M.A., Abdalla M.G.A., Tsujimoto H. (2017). Genetic variation in heat tolerance-related traits in a population of wheat multiple synthetic derivatives. Breed. Sci..

[45] Gorafi Y.S.A., Kim J.S., Elbashir A.A.E., Tsujimoto H. (2018). A population of wheat multiple synthetic derivatives: An effective platform to explore, harness and utilize genetic diversity of *Aegilops tauschii* for wheat improvement. Theor. Appl. Genet..

